# Genetically encoded photocross-linkers determine the biological binding site of exendin-4 peptide in the N-terminal domain of the intact human glucagon-like peptide-1 receptor (GLP-1R)

**DOI:** 10.1074/jbc.M117.779496

**Published:** 2017-03-10

**Authors:** Cassandra Koole, Christopher A. Reynolds, Juan C. Mobarec, Caroline Hick, Patrick M. Sexton, Thomas P. Sakmar

**Affiliations:** From the ‡ Laboratory of Chemical Biology and Signal Transduction, The Rockefeller University, New York, New York 10065,; ¶ Drug Discovery Biology, Monash Institute of Pharmaceutical Sciences, Monash University, Parkville, Victoria 3052, Australia, and; the § School of Biological Sciences, University of Essex, Wivenhoe Park, Colchester CO4 3SQ, United Kingdom

**Keywords:** G protein-coupled receptor (GPCR), molecular modeling, mutagenesis, peptide hormone, structural biology, structure-function, glucagon-like peptide-1 receptor (GLP-1R), genetic code expansion, photocross-linking, unnatural amino acid

## Abstract

The glucagon-like peptide-1 receptor (GLP-1R) is a key therapeutic target in the management of type II diabetes mellitus, with actions including regulation of insulin biosynthesis and secretion, promotion of satiety, and preservation of β-cell mass. Like most class B G protein-coupled receptors (GPCRs), there is limited knowledge linking biological activity of the GLP-1R with the molecular structure of an intact, full-length, and functional receptor·ligand complex. In this study, we have utilized genetic code expansion to site-specifically incorporate the photoactive amino acid *p*-azido-l-phenylalanine (azF) into N-terminal residues of a full-length functional human GLP-1R in mammalian cells. UV-mediated photolysis of azF was then carried out to induce targeted photocross-linking to determine the proximity of the azido group in the mutant receptor with the peptide exendin-4. Cross-linking data were compared directly with the crystal structure of the isolated N-terminal extracellular domain of the GLP-1R in complex with exendin(9–39), revealing both similarities as well as distinct differences in the mode of interaction. Generation of a molecular model to accommodate the photocross-linking constraints highlights the potential influence of environmental conditions on the conformation of the receptor·peptide complex, including folding dynamics of the peptide and formation of dimeric and higher order oligomeric receptor multimers. These data demonstrate that crystal structures of isolated receptor regions may not give a complete reflection of peptide/receptor interactions and should be combined with additional experimental constraints to reveal peptide/receptor interactions occurring in the dynamic, native, and full-length receptor state.

## Introduction

G protein-coupled receptors (GPCRs)^4^ are integral membrane proteins that mediate transmembrane (TM) signaling in response to a diverse range of extracellular stimuli, including neurotransmitters, hormones, odorants, and light (1). Accounting for as much as 2% of the human genome (2), GPCRs have a fundamental role in almost all physiological functions and as such have become key therapeutic targets in the treatment of many disease states. Although almost one-third of marketed drugs act on GPCRs (3), very few target the small subgroup of peptide-activated class B “secretin-like” receptors. Despite this, class B GPCRs have been validated for the treatment of a number of disease conditions, including depression and anxiety (corticotropin-releasing factor (CRF) receptors), osteoporosis (calcitonin and parathyroid hormone receptors), and metabolic disorders (glucagon-like peptide-1 (GLP-1), gastric inhibitory polypeptide (GIP), glucagon (GCG), and amylin receptors) (4). To fully unlock the therapeutic potential of this receptor class, it is essential to understand not only the pharmacology of these receptor systems in greater detail but also the molecular and structural dynamics induced by peptide/receptor interactions that underlie their functional outcomes.

Until recently, structural information for class B GPCRs was limited to NMR and X-ray structures of isolated N-terminal extracellular domains (ECDs) for several family members (5–9), with homology modeling and mutagenesis data used to estimate the core TM-spanning region (10). Recent reports of inactive TM crystal structures of the GCG receptor (GCGR) (11, 12) and CRF1 receptor (CRF1R) (13) have since provided significant improvement in understanding class B GPCRs, revealing distinct orientations in TM bundle architecture compared with class A GPCRs. Although crystallography is a powerful technique in establishing receptor structure, this platform only identifies static snapshots of highly dynamic entities. Furthermore, none of the reported class B structures are of full-length receptor proteins; N-terminal structures lack the entire TM region, and TM structures have substantial N- and C-terminal truncations. As with all documented GPCR crystal structures, both GCGR and CRF1R were purified with large bulky modifications (T4L for CRF1R and BRIL or T4L for GCGR), multiple thermostabilizing mutations, and were crystallized in the presence of small molecule antagonists (11–13). Consequently, the information ascertained from these structures may not give a complete account of native receptor configuration or peptide/receptor interactions required to achieve the “active” receptor conformations that drive intracellular signaling.

Beyond crystallography, investigation of peptide/receptor interactions has principally involved receptor mutagenesis and incorporation of photolabile probes into peptides (14). While providing valuable insights, both strategies have limited interpretability due to structural perturbations inflicted on the receptor and peptide, respectively. More recently, genetic incorporation of photoactive unnatural amino acids into receptor proteins at putative peptide/receptor interaction points has gained traction, because of high tolerability and low impact on structural integrity (15–17). Furthermore, direct identification of receptor binding contacts can be achieved by exploiting the photocross-linking properties of the amino acid, as demonstrated for several receptor proteins (15, 16, 18–22), and most recently the serotonin transporter (23). Importantly, this method can be conducted in mammalian cells (24–27), mimicking the native receptor environment.

The subject of this study, GLP-1R, plays a fundamental role in controlling post-prandial insulin secretion upon interaction with its cognate ligand, GLP-1, or exogenous peptides, such as exendin-4 (28). Additionally, with a broad range of actions, including promotion of insulin synthesis, decreasing GCG production, preservation of pancreatic β-cell mass, decreasing appetite, and gastric emptying, as well as cardio- and neuroprotective functions (29, 30), the GLP-1R is an ideal target for development of treatments for type II diabetes mellitus and obesity. As such, comprehensive knowledge of peptide/receptor interactions within this receptor system is vital in the development of superior therapeutics to manage the condition.

In this study, we have successfully incorporated the photoactive amino acid *p*-azido-l-phenylalanine (azF) into the human GLP-1R at N-terminal ECD sites reported to interact with peptide agonists (Table 1). Using the photocross-linking feature of azF, we have mapped the principal binding region between the GLP-1R N terminus and the exogenous peptide exendin-4 in live cells expressing intact GLP-1R, and we have used these data to generate a detailed model of the GLP-1R·exendin-4 complex. We observe that not all GLP-1R/exendin-4 interactions or proximity estimates documented in the literature are consistent with cross-linking data, demonstrating that crystal structures of isolated receptor regions (N-terminal or TM domains) may not give a true reflection of peptide/receptor interactions occurring in the dynamic, native, and full-length receptor state. Expansion of this technology to include other GLP-1R peptide ligands may allow for detection of subtle differences in receptor/peptide interactions and binding domains, aiding our understanding of the complex molecular mechanisms underlying the relationship between peptide binding and biased signaling at the GLP-1R.

## Results

### Selection of human GLP-1R residues for amber mutagenesis and unnatural amino acid incorporation

**Figure 1.**
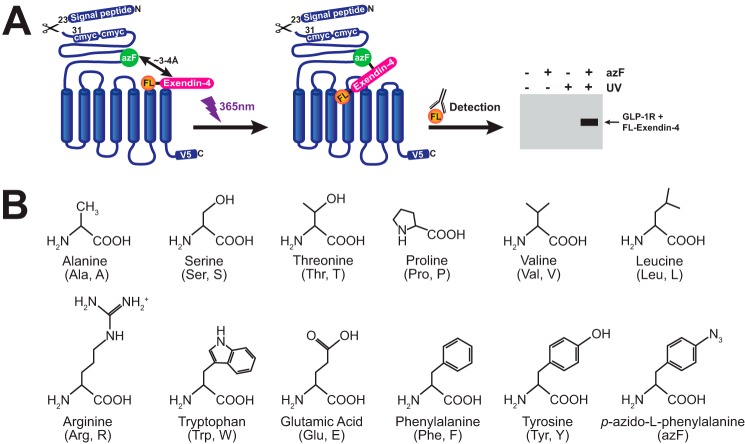
**Schematic of azF-mediated photocross-linking at the GLP-1R and amino acids mutated to azF in this study.** *A,* all experiments were conducted on the human GLP-1R containing an N-terminal double c-Myc epitope following the signal peptide (residues 1–23), and a C-terminal V5 epitope. AzF is incorporated at site-specific in-frame amber (TAG) mutations using a heterologous cell system and the amber codon suppression machinery detailed in Ye *et al.* (24). Cells expressing azF-incorporated GLP-1Rs are incubated to equilibrium with FL-labeled exendin-4 (FL-exendin-4), followed by exposure to UV light (365 nm). Photoactivation of azF promotes formation of a covalent bond with primary amines or aliphatic hydrogens lying within its proximity (up to 3–4 Å) (15, 18), allowing the receptor to capture the peptide. The receptor·peptide complex can then be immunologically detected using FL-specific Abs at a size that is the sum of both components. *B,* schematic of the amino acids mutated to azF in the human GLP-1R, with three-letter and single-letter nomenclature in *parentheses*.

To establish the peptide/receptor interface within the N-terminal GLP-1R ECD, the unnatural amino acid azF was site-specifically integrated into the human GLP-1R using the amber codon suppression technology in a recombinant HEK293T cell system. This strategy exploits the ability of azF to covalently link an interacting partner within close proximity (up to 3–4 Å) upon UV irradiation, and in this application a ligand·receptor complex can be captured (Fig. 1*A*). Amino acid residues of the GLP-1R chosen for amber mutagenesis and subsequent azF incorporation encompassed both polar and non-polar, charged, and aromatic amino acids (Fig. 1*B*), and they were based on documented interactions between peptide agonists and the N-terminal GLP-1R ECD through NMR and X-ray crystallography (5, 6), as well as proximity analysis using *p*-benzoyl-l-phenylalanine (Bpa)-labeled GLP-1 peptides (Table 1) (31, 32).

**Table 1:** **AzF-substituted residues of the human GLP-1R studied for cross-linking and their previously documented interactions with peptide** Interactions are reported from isolated N-terminal domain crystal structures and previous cross-linking data. Equivalent residues for class B receptors are based on homology alignment from Parthier *et al.* (9).

GLP-1R residue	Reported interaction/s*^a^*	Reported interactions in class B GPCR equivalent residues	Refs.
Val-30	Ligand recognition/specificity		5
		Gln-30 GIPR interacts with GIP	9
Ser-31	Ligand recognition/specificity		5
Leu-32	Ligand recognition/specificity		5
	Ex(9–39) interaction point (F^22^)		
	GLP-1 interaction point (A^24^, A^25^, and F^28^)		6
		Ala-32 GIPR interacts with GIP	9
Trp-33	No documented interaction		
Thr-35	Ex(9–39) interaction point (F^22^)		5
		Leu-35 GIPR interacts with GIP	9
Val-36	Ex(9–39) interaction point (F^22^)		5
		Tyr-36 GIPR interacts with GIP	9
Trp-39	Ex(9–39) interaction point (F^22^)		5
		Trp-36 GCGR interacts with GCG	12
	Conserved across GCG class B GPCRs	Trp-39 GIPR interacts with GIP	9
Arg-40	No documented interaction		
Tyr-42	No documented interaction		5
	Conserved across GCG class B GPCRs		
Phe-61	No documented interaction		
Glu-68	Ex(9–39) interaction point (S^32^)		5
		Met-67 GIPR interacts with GIP	9
Tyr-69	Ex(9–39) interaction point		5
	GLP-1 interaction point (V^33^)		6
		Tyr-68 GIPR interacts with GIP	9
Ala-70	No documented interaction		
Phe-80	No documented interaction		
Tyr-88	Ex(9–39) interaction point		5
		Tyr-87 GIPR interacts with GIP	9
Leu-89	Ex(9–39) interaction point		5
		Leu-88 GIPR interacts with GIP	9
Pro-90	Ex(9–39) interaction point (V^19^ and I^23^)		5
		Pro-89 GIPR interacts with GIP	9
Trp-91	Ex(9–39) interaction point (V^19^ and I^23^)		5
		Trp-90 GIPR interacts with GIP	9
Tyr-101	No documented interaction		
Phe-103	No documented interaction		
Ser-116	No documented interaction		
Ser-117	No documented interaction		
	*N*-Glycosylation recognition domain		49
Leu-118	No documented interaction		
Pro-119	Putative GLP-1 interaction point		6
Arg-121	Ex(9–39) interaction point (K^27^)		5
	GLP-1 interaction point (V^33^)		6
		Arg-113 GIPR interacts with GIP	9
Leu-123	Ex(9–39) interaction point		5
	GLP-1 interaction point (V^33^)		6
		His-115 GIPR interacts with GIP	9
Ser-124	No documented interaction		
Glu-125	Proximal to GLP-1 peptide (cross-linking) (Bpa^35^)		31
Glu-127	Ex(9–39) interaction point (K^27^ and E^24^)		5
	No documented GLP-1 interaction		6
Arg-134	No documented interaction		
Ser-135	No documented interaction		
Glu-138	No documented interaction		

*^a^* Peptide residue number is denoted in superscript following single code amino acid.

Consideration was also given to interacting residues reported in related GIP receptor (GIPR) (9) and GCGR (12) crystal structures (Table 1). To validate the methodology, several GLP-1R residues not documented to interact with peptide, and distal to the predicted peptide-binding domain, were also selected for azF incorporation.

### AzF mutants are expressed at the cell surface in the presence of azF supplementation

**Figure 2.**
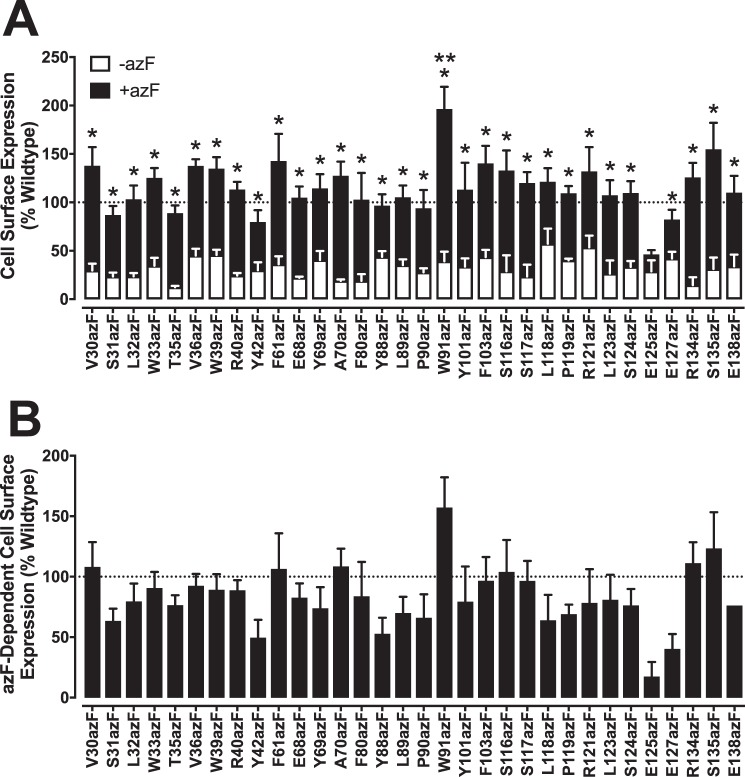
**Cell-surface expression profiles of azF-incorporated GLP-1R amber mutants.** *A,* cell-surface expression of each of the human GLP-1R amber mutants transiently transfected into HEK293T cells in the absence (*open bars*) and presence (*closed bars*) of 0.5 mm azF, as determined through Ab detection of the N-terminal double c-Myc epitope. *B,* azF-dependent cell-surface expression of each of the human GLP-1R amber mutants, corrected for receptor expression observed in the absence of azF. Data are normalized to the cell-surface expression measured for wild-type human GLP-1R, transfected at one-tenth that of mutants (100%), and errors were propagated from both −azF and +azF. Changes in cell-surface expression for each mutant in the absence and presence of azF was determined using an unpaired *t* test, and statistical significance was accepted as *p* < 0.05 (*). Changes in azF-dependent cell-surface expression in comparison with wild-type control was determined by one-way analysis of variance and Dunnett's post-test, and statistical significance was accepted as *p* < 0.05 (**). All data are mean ± S.E. of three to eight independent experiments, conducted in duplicate.

Cell-surface expression of azF-incorporated human GLP-1R amber mutants was determined through immunological detection of the receptor N-terminal double c-Myc epitope (Fig. 2*A*). Supplementation of transfected amber mutants with azF resulted in cell-surface receptor expression of mutants that was statistically comparable with that of the wild-type receptor control expressed at one-tenth that of mutants, with the exception of W91azF that had significantly elevated expression and E125azF that was poorly expressed (Fig. 2*A* and Table 2). In all cases, low level cell-surface expression of amber mutants was detected in the absence of azF supplementation, likely resulting from incomplete efficiency in amber codon suppression, instead incorporating endogenous Tyr at the amber codon site during ribosomal elongation of the receptor product (Fig. 2*A* and Table 2) (24). With the exception of E125azF, the level of cell-surface expression of each receptor mutant in the absence of azF was statistically lower than that observed in the presence of azF, indicating successful incorporation of the azF residue (Table 2). As noted previously (33), antibody (Ab) detection of the N-terminal c-Myc epitope does not distinguish between different receptor products, and therefore in the presence of azF, a mixed population of both Tyr- and azF-incorporated receptors are likely being detected at the cell surface. To account for this, cell-surface expression of each receptor mutant was corrected for that measured in the absence of azF, allowing quantification of azF-dependent receptor expression at the cell surface (Fig. 2*B*). Despite the overall reduction in cell-surface expression as a result of this correction, all mutant receptors maintained statistically comparable expression to wild-type GLP-1R (Fig. 2*B* and Table 2), although azF-substituted Tyr-42, Tyr-88, Glu-125, and Glu-127 all exhibited strong trends toward lower comparative expression.

**Table 2:** **Summary of the effects of azF incorporation into the human GLP-1R** HEK293T cells transiently expressing human GLP-1R wild-type or amber mutants in the absence (−) and presence (+) of 0.5 mm azF are shown. Total cell-surface expression was determined through Ab detection of the N-terminal c-Myc epitope label, with data expressed as a maximum of wild-type human GLP-1R expression. AzF-dependent cell-surface expression was determined through baseline correction to the expression observed for each mutant receptor in the absence of azF, with data expressed as a maximum of wild-type human GLP-1R expression and errors propagated from both −azF and +azF conditions. Exendin-4-mediated cAMP data were analyzed using a three-parameter logistic equation as defined in Equation 1. pEC_50_ values represent the negative logarithm of the concentration of agonist that produces half the maximal response. *E*_max_ represents the maximal response normalized to that elicited by 10 μm forskolin. Relative cross-linking of FL-Ex4 at receptor mutants was determined as a measure of optical density, normalized to the respective controls on the same blot. Values are expressed as mean ± S.E. of three to eight independent experiments, conducted in duplicate or triplicate. Cell surface expression of each mutant receptor in the absence and presence of azF supplementation was analyzed using an unpaired *t* test, and statistical significance was accepted as *p* < 0.05 (bold, *). All other data were analyzed with one-way analysis of variance and Dunnett's post-test, and statistical significance accepted as *p* < 0.05 in comparison with wild-type control (bold, **). ND, not detected.

	Cell surface expression (% wild type)		azF-dependent cell-surface expression (% wild type)	cAMP (Exendin-4) (+azF)		Specific cross-linking (% Y69azF)
	−azF	+azF	+azF	pEC_50_	*E*_max*^a^*_	
Wild type*^b^*	99.9 ± 0.1	100.0 ± 0.1	100.0 ± 0.1	10.8 ± 0.1	64.5 ± 2.2	ND
V30azF	29.6 ± 7.0	137.5 ± 19.6*	107.8 ± 20.8	11.2 ± 0.3	45.5 ± 3.3**	143.5 ± 27.7
S31azF	23.4 ± 4.0	86.6 ± 9.6*	63.2 ± 10.4	11.1 ± 0.2	54.1 ± 3.0**	74.1 ± 12.7
L32azF	23.6 ± 3.5	102.9 ± 14.6*	79.3 ± 15.0	11.1 ± 0.3	48.0 ± 3.1**	194.1 ± 46.1
W33azF	34.5 ± 8.3	124.9 ± 10.6*	90.4 ± 13.5	11.4 ± 0.1	66.4 ± 2.2	115.2 ± 41.2
T35azF	12.3 ± 1.5	88.5 ± 8.3*	76.3 ± 8.4	9.5 ± 0.2**	59.2 ± 3.6	ND
V36azF	45.1 ± 7.0	137.4 ± 7.2*	92.3 ± 10.0	11.4 ± 0.1	63.8 ± 2.3	78.7 ± 40.3
W39azF	45.7 ± 5.4	134.6 ± 12.0*	88.9 ± 13.2	10.6 ± 0.2	61.2 ± 2.7	ND
R40azF	24.5 ± 2.5	113.0 ± 8.2*	88.6 ± 8.5	11.5 ± 0.1	62.3 ± 1.9	80.9 ± 26.5
Y42azF	30.0 ± 8.1	79.3 ± 12.6*	49.4 ± 15.0	10.6 ± 0.2	37.4 ± 2.2**	ND
F61azF	36.1 ± 8.3	142.4 ± 28.5*	106.3 ± 29.7	11.3 ± 0.2	67.5 ± 2.6	6.2 ± 4.8
E68azF	22.1 ± 1.2	104.6 ± 11.9*	82.5 ± 12.0	11.4 ± 0.2	70.9 ± 2.6	31.2 ± 6.8
Y69azF	40.5 ± 9.4	114.1 ± 15.0*	73.7 ± 17.7	10.8 ± 0.3	29.2 ± 2.0**	100
A70azF	18.9 ± 1.5	127.3 ± 14.8*	108.3 ± 14.9	9.1 ± 0.1**	69.1 ± 2.8	ND
F80azF	19.0 ± 7.0	102.6 ± 27.8*	83.6 ± 28.6	11.3 ± 0.2	69.0 ± 2.8	53.1 ± 13.4
Y88azF	43.7 ± 6.0	96.3 ± 12.0*	52.6 ± 13.4	10.6 ± 0.2	28.2 ± 1.7**	ND
L89azF	35.3 ± 5.8	105.0 ± 12.3*	69.7 ± 13.6	9.6 ± 0.1**	70.4 ± 2.6	ND
P90azF	27.8 ± 4.2	93.7 ± 19.1*	65.9 ± 19.6	9.9 ± 0.2**	51.1 ± 4.0**	ND
W91azF	39.1 ± 9.9	196.2 ± 23.1*,**	157.0 ± 25.2	11.5 ± 0.2	64.4 ± 3.6	8.5 ± 5.0
Y101azF	43.5 ± 7.5	112.9 ± 28.0*	79.2 ± 29.3	11.5 ± 0.2	62.1 ± 2.3	36.9 ± 17.1
F103azF	45.9 ± 8.2	139.9 ± 18.4*	96.5 ± 19.8	11.4 ± 0.2	55.8 ± 2.1	51.3 ± 17.2
S116azF	28.9 ± 16.5	132.6 ± 21.0*	103.7 ± 26.7	11.4 ± 0.1	69.3 ± 1.6	16.3 ± 6.9
S117azF	23.5 ± 12.2	119.8 ± 11.6*	96.3 ± 16.8	11.1 ± 0.2	64.2 ± 2.8	52.5 ± 17.7
L118azF	57.3 ± 15.6	121.1 ± 14.3*	63.8 ± 21.2	11.5 ± 0.1	72.4 ± 1.8	99.6 ± 24.5
P119azF	40.1 ± 1.8	109.0 ± 7.8*	68.9 ± 8.0	11.4 ± 0.1	71.7 ± 1.5	ND
R121azF	53.6 ± 12.0	131.7 ± 25.4*	78.1 ± 28.1	10.0 ± 0.1	71.6 ± 1.9	ND
L123azF	26.4 ± 13.6	107.1 ± 15.8*	80.7 ± 20.9	11.2 ± 0.3	19.9 ± 1.5**	145.3 ± 30.8
S124azF	33.4 ± 6.0	109.4 ± 12.5*	76.0 ± 13.9	11.4 ± 0.1	72.2 ± 1.9	ND
E125azF	28.8 ± 11.3	46.0 ± 4.7	17.2 ± 12.3	10.3 ± 0.1	66.4 ± 2.5	ND
E127azF	42.0 ± 7.0	82.0 ± 10.3*	40.1 ± 12.5	10.8 ± 0.1	76.8 ± 2.0	128.8 ± 59.2
R134azF	14.4 ± 8.2	125.4 ± 15.4*	111.0 ± 17.4	11.1 ± 0.3	40.5 ± 3.4**	102.4 ± 29.2
S135azF	31.2 ± 11.9	154.5 ± 27.7*	123.2 ± 30.1	11.5 ± 0.1	75.7 ± 2.6	48.4 ± 8.1
E138azF	34.0 ± 12.1	109.8 ± 21.3*	75.8 ± 21.3	11.0 ± 0.3	39.2 ± 2.7**	ND

*^a^* The *E*_max_ values are represented as the maximal response normalized to that elicited by 10 μm forskolin.

*^b^* The wild-type human GLP-1R was transfected at one-tenth that of mutant receptors.

### Functional impact of azF incorporation into the human GLP-1R

Amber mutagenesis and incorporation of unnatural amino acids such as azF have been observed to be well tolerated at most receptor residues, at least in class A GPCR applications (24, 25, 34). To determine whether this observation translated into class B GPCRs, azF-incorporated GLP-1Rs were examined for their ability to generate a cAMP response, a typical functional readout directly correlated with insulin secretion in a physiological setting (30). Consistent with that previously reported (35), exendin-4 stimulated a robust cAMP response at the wild-type GLP-1R, with a pEC_50_ value of 10.8 ± 0.1 (Table 2). In the presence of azF, most mutant receptors were able to generate an exendin-4-mediated cAMP response, with functional affinity statistically comparable with wild-type control (pEC_50_, Table 2 and supplemental Fig. S1). The exceptions to this were T35azF, A70azF, L89azF, and P90azF that had 20-, 50-, 16-, and 8-fold decreases in potency, respectively, relative to wild-type control (Table 2). The loss of functional affinity did not correlate with a decrease in total cell-surface expression (Fig. 2*A* and Table 2), suggesting that the attenuation in function may be a result of decreased ligand-binding affinity and/or functional coupling at these receptor variants. The impact of azF incorporation into the GLP-1R was more readily observed in functional efficacy, with significant decreases in *E*_max_ for exendin-4 at V30azF, S31azF, L32azF, Y42azF, Y69azF, Y88azF, P90azF, L123azF, R134azF, and E138azF (Table 2 and supplemental Fig. S1). Despite the attenuated responses, these mutant receptors maintained statistically similar expression to wild type, suggesting these residues are functionally or structurally important. Notably, the functional response measured is likely a composite of both azF- and Tyr-incorporated receptors, as discussed above, and therefore deducing the population of azF-incorporated receptors specific to the cAMP response is not possible. Additionally, the method of cAMP detection in this system is amplified such that even in the absence of azF the ability of mutant receptors to generate an exendin-4-mediated cAMP response is not always dissimilar to that in the presence of azF (data not shown). Nonetheless, these data demonstrate that in most cases mutation to either azF or Tyr is generally well tolerated by the receptor protein.

### Functional impact of fluorescein (FL) incorporation into GLP-1R peptide agonists

With the lack of robust Abs highly specific to any GLP-1R peptide ligand, detection relies on conjugation or incorporation of measurable probes into the peptide itself, such as biotin, radioisotopes, or fluorophores. GLP-1 and GLP-1-related peptides are sensitive to modification, particularly in the N-terminal domain, which is required for efficient GLP-1R activation. Examples of this include exendin-4(1–39), a high affinity agonist *versus* exendin(9–39), a high affinity antagonist, and GLP-1(7–36)NH_2_, a high affinity agonist *versus* GLP-1(9–36)NH_2_, a low affinity partial agonist (36). These data also indicate that N-terminally modified exendin-4 retains efficient interaction with the receptor despite loss of functional efficacy.

Radioisotopic labeling is a preferred method for many peptide ligand applications, due to its ready detection and flexibility in labeling via Tyr residues or Bolton-Hunter-mediated labeling of amides. However, the process of radiolabeling and subsequent utilization of radiolabeled products in an experimental setting is cumbersome, expensive, and hazardous. Consequently, we elected to employ peptide ligands conjugated to FL, a small yet intensely fluorescent probe that can also be detected immunologically through use of Abs. FL was conjugated to the exendin-4 peptide at the N terminus (FL-Ex4) or at amino acid positions Trp-25 (FL(W25)-Ex4), Lys-27 (FL(K27)-Ex4), or Arg-20 (FL(R>K20)-Ex4, subsequently referred to as FL(K20)-Ex4), the latter being synonymous with the position of hexadecanoic acid (C_16_H_32_O_2_) modification in the GLP-1 mimetic, liraglutide (37). Each modified exendin-4 analogue was assessed for cAMP response at the wild-type human GLP-1R (Table 3 and supplemental Fig. S2). Compared with native exendin-4 (pEC_50_ 11.0 ± 0.1), addition of the FL moiety to the N terminus of the peptide (FL-Ex4) significantly attenuated the cAMP response, resulting in an ∼500-fold reduction in potency (Table 3), likely as a result of disruption to the positioning of the peptide N terminus within the receptor core. Significant reductions in potency were also observed for FL(K20)-Ex4 and FL(K27)-Ex4, albeit the fold reduction was not as severe as that for N-terminal FL addition (50- and 16-fold, respectively), whereas FL(W25)-Ex4 did not significantly deviate from the unmodified peptide (Table 3).

**Table 3:** **Effect of FL incorporation into exendin-4 on cAMP accumulation in HEK293T cells transiently expressing the human GLP-1R** Data were analyzed using a three-parameter logistic equation as defined in Equation 1. pEC_50_ values represent the negative logarithm of the concentration of agonist that produces half the maximal response. *E*_max_ represents the maximal response normalized to that elicited by 10 μm forskolin. Values are expressed as mean ± S.E. of three to six independent experiments, conducted in duplicate or triplicate. Data were analyzed with one-way analysis of variance and Dunnett's post-test, and statistical significance accepted as *p* < 0.05 in comparison to the unmodified peptide (bold, *).

	pEC_50_	*E*_max_
*Ex4*	11.0 ± 0.1	64.4 ± 1.9
*FL-Ex4*	8.3 ± 0.2*	44.6 ± 4.6*
*FL(W25)-Ex4*	10.5 ± 0.1	66.5 ± 2.0
*FL(K20)-Ex4*	9.3 ± 0.1*	60.9 ± 2.1
*FL(K27)-Ex4*	9.8 ± 0.2*	66.4 ± 6.0

### Mapping the exendin-4-binding site in the human GLP-1R N terminus using azF-mediated photocross-linking in live cells

**Figure 3.**
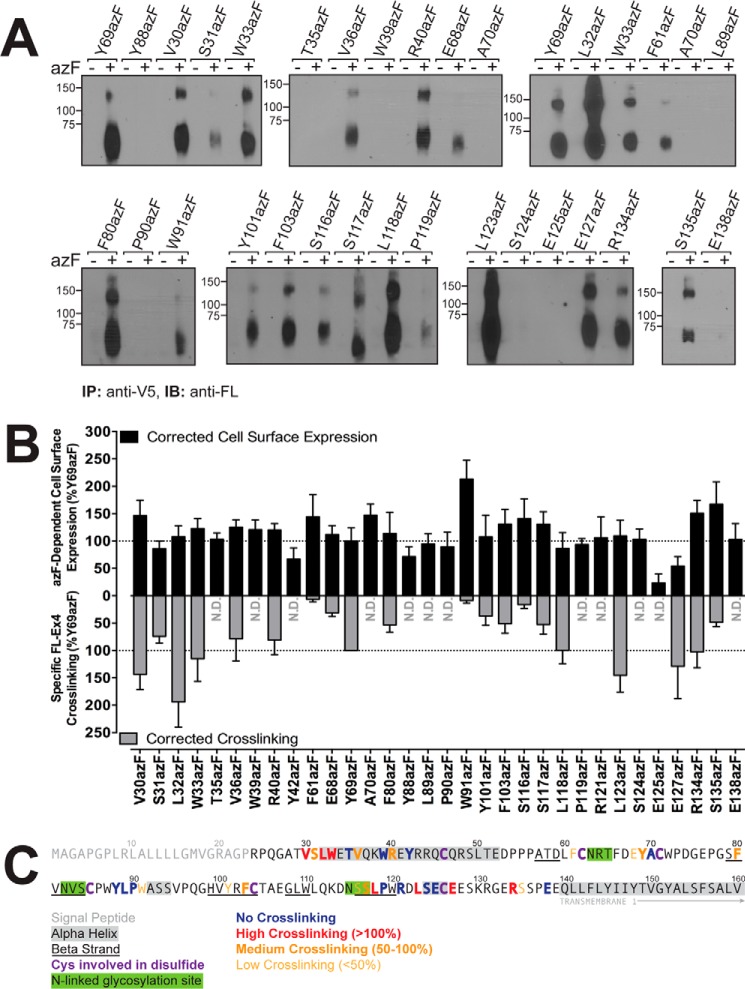
**Mapping the exendin-4-binding site within the human GLP-1R N terminus using azF-mediated cross-linking.** *A,* photocross-linking of azF-incorporated GLP-1Rs with FL-Ex4. HEK293T cells transiently expressing each of the human GLP-1R amber mutants in the absence (−) and presence (+) of 0.5 mm azF were incubated with 10 nm FL-Ex4 followed by exposure to UV light for 2 min at 4 °C. Whole-cell lysates were then immunoprecipitated (*IP*) using an anti-V5 Ab to isolate full-length GLP-1Rs, and products were resolved by SDS-PAGE. Bands detected with the anti-FL Ab (immunoblot, *IB*) identify receptor positions at which azF covalently captures the FL-Ex4 ligand. Non-cross-linked FL-Ex4 was not detected (∼4.5 kDa). Data are representative of three to eight independent experiments. *B,* comparison between azF-dependent cell-surface expression of human GLP-1R amber mutants and relative photocross-linking with FL-Ex4. azF-dependent cell-surface expression of human GLP-1R amber mutants transiently transfected into HEK293T cells in the presence of 0.5 mm azF (*closed bars,* above *x* axis) was determined through Ab detection of the N-terminal double c-Myc epitope. Data are corrected for cell-surface expression measured in the absence of azF, normalized to the wild-type GLP-1R control transfected at one-tenth that of mutants, and subsequently normalized to the azF-dependent cell-surface expression measured for Y69azF (100%, internal control). Relative cross-linking of FL-Ex4 at human GLP-1R amber mutants transiently transfected into HEK293T cells in the presence of 0.5 mm azF (*gray bars,* below *x* axis) was determined as a measure of optical density of the band corresponding to the monomeric receptor·ligand complex (∼65 kDa), normalized to the respective wild-type control on the same blot, and subsequently normalized to the optical density measured for Y69azF on the same blot (100%, internal control). All data are mean ± S.E. of three to eight independent experiments. *N.D.,* not detected. *C,* summary of human GLP-1R N-terminal ECD residues determined in this study to cross-link with FL-Ex4.

As detailed above, azF incorporation at site-specifically introduced amber codons is not 100% efficient, allowing incorporation of endogenous Tyr (24). Nonetheless, this does not impact on the application of the targeted photocross-linking method, as only azF-incorporated receptors have the ability to covalently capture close proximity partners upon UV activation. Cells transiently expressing human GLP-1R with site-specifically incorporated azF were equilibrated with 100-fold the functional affinity (pEC_50_; as determined through cAMP accumulation) of each FL-conjugated exendin-4 peptide and irradiated with UV light (365 nm). Resultant azF-mediated cross-links between peptide and receptor were determined through immunodetection of the FL moiety occurring at the size of the peptide·receptor complex (∼65 kDa (monomeric receptor) and ∼150 kDa (dimeric receptor)). Interpretation of cross-linking profiles for each of FL(K20)-Ex4, FL(W25)-Ex4, and to a lesser extent FL(K27)-Ex4 to azF-incorporated GLP-1Rs was problematic due to weak signal obtained when probing for FL (data not shown), potentially because of formation of a peptide·receptor complex that buries the FL moiety within a region inaccessible, or only partially accessible, by the FL-directed Ab. For these analogues, the location of FL incorporation within the exendin-4 peptide also likely occurs in a region of the peptide that binds the receptor N terminus, impacting accurate interpretation of interactions between exendin-4 and the GLP-1R N terminus using the cross-linking method (supplemental Fig. S3). For these reasons, the FL(K20)-Ex4 and FL(W25)-Ex4 analogues were not pursued further for photocross-linking, whereas FL(K27)-Ex4 was limited to confirmatory studies. Despite the lower functional affinity observed for FL-Ex4 at the GLP-1R compared with the other exendin-4 analogues (Table 3), this peptide robustly cross-linked to multiple azF-incorporated GLP-1Rs in a UV-dependent manner (Fig. 3*A*). As observed previously (16, 18), there was no cross-linking between receptor and peptide in the absence of azF supplementation or in the absence of UV exposure (Fig. 3*A*, data not shown). Homologous competition with increasing concentrations of unlabeled exendin-4 almost completely abolished formation of the GLP-1R·FL-Ex4 complex, whereas the preformed, UV-cross-linked GLP-1R·FL-Ex4 complex was not disrupted by excess unlabeled exendin-4, suggesting that FL-Ex4 occupies a similar binding cavity to unlabeled exendin-4, as well as confirming the covalent nature of azF-mediated cross-links (supplemental Fig. S4, *A* and *B* and data not shown). Interestingly, cells expressing the wild-type GLP-1R sporadically displayed UV-mediated nonspecific cross-linking with FL-Ex4 in both the presence and absence of azF (supplemental Fig. S4, *A* and *B*). These phenomena were not isolated artifacts of FL-Ex4, as nonspecific cross-linking of FL(K27)-Ex4 was also observed, albeit the strength of signal was proportionally lower (supplemental Fig. S4, *C* and *D*). In both cases, the level of cross-linking observed at the wild-type receptor was consistently lower than that of azF-incorporated receptors, despite similar levels of expression (Fig. 2 and Table 2). Subsequent determination and quantification of specific FL-Ex4 cross-linking to azF-incorporated GLP-1Rs therefore involved correction for background signal occurring at the wild-type GLP-1R. To account for variation across individual experiments, data were also normalized to the level of cross-linking observed with Y69azF that was run as a control in each experiment. Of the residues analyzed in this study, robust cross-linking was observed between FL-Ex4 and GLP-1R mutants V30azF, S31azF, L32azF, W33azF, V36azF, R40azF, E68zF, Y69azF, F80azF, Y101azF, F103azF, S117azF, L118azF, L123azF, E127azF, R134azF, and S135azF (Fig. 3 and Table 2), although weak and inconsistent cross-linking was observed for S116azF in some experiments (Fig. 3 and Table 2). Weak or no interactions were identified between FL-Ex4 and GLP-1R mutants T35azF, W39azF, Y42azF, F61azF, A70azF, Y88azF, L89azF, P90azF, W91azF, P119azF, R121azF, S124azF, E125azF, and E138azF (Fig. 3 and Table 2). The relative level of cross-linking, as determined through densitometry analysis, did not directly correlate with azF-dependent expression of mutant receptors; for example, S135azF had low level cross-linking with FL-Ex4 but trended toward increased expression compared with the wild-type receptor, and E127azF was highly efficient in cross-linking with FL-Ex4 but had low expression relative to the wild-type control (Figs. 2*B* and 3*B*). Of note, most mutants that displayed high levels of cross-linking exhibited decreases in exendin-4-mediated cAMP responses, including V30azF, L32azF, Y69azF, L123azF, and R134azF that was unrelated to their level of cell-surface receptor expression (Fig. 3*B* and supplemental Fig. S1), although W33azF and E127azF were exceptions to this trend.

### Comparison of the GLP-1R-FL-Ex4 cross-linking profile with the isolated GLP-1R N-terminal exendin(9–39) crystal structure

**Figure 4.**
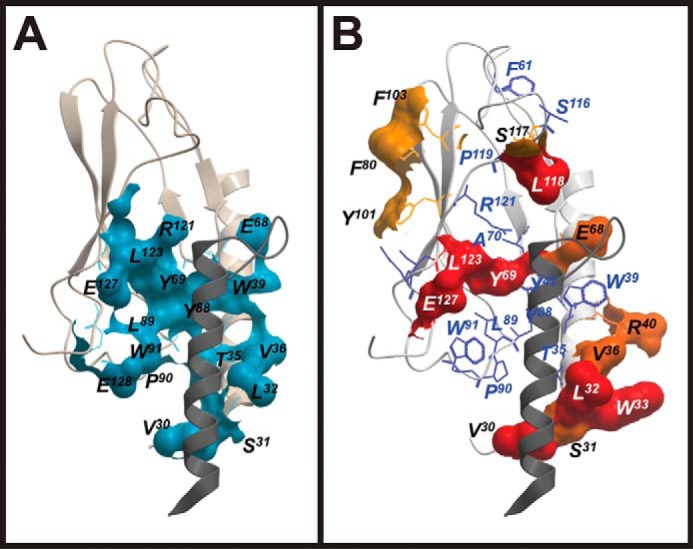
**Comparison of the interaction surface of the human GLP-1R N terminus with exendin-4, as identified by crystallography and azF-mediated cross-linking.** *A,* crystal structure of the isolated N-terminal ECD of the human GLP-1R in complex with exendin-4 (PDB code 3C59). The interaction surface of the receptor (within 5 Å of exendin-4) is shown in *blue*; the backbone of the receptor N terminus is shown as a *pink ribbon,* and the backbone of the exendin-4 peptide as a *dark gray ribbon. B,* azF-substituted residues of the full-length human GLP-1R mapped to the crystal structure of the isolated human GLP-1R N terminus in complex with exendin-4 (PDB code 3C59). Residues cross-linking to FL-Ex4 are displayed in surface representation, colored according to strength of cross-linking (*red*, strong; *dark orange*, moderate; *light orange*, weak). azF-substituted residues that did not cross-link to FL-Ex4 are shown in *wire* representation, colored *navy blue*. The backbone of the receptor N terminus is colored in *off-white ribbon,* and the backbone of the exendin-4 peptide in *dark gray ribbon*.

**Figure 5.**
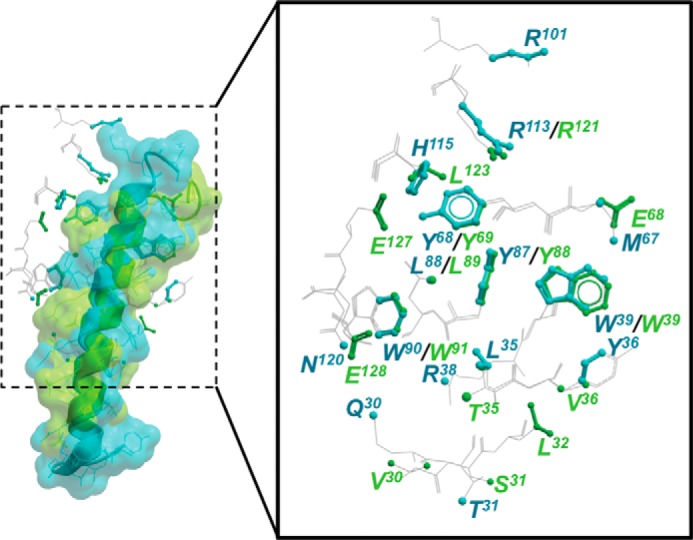
**GIP and exendin-4 occupy a similar groove and make conserved contacts with their respective receptors in X-ray crystal structures of the isolated N-terminal ECDs.** *Left panel,* GIP (*blue*) and exendin-4 (*green*) are shown as *ribbon* and *wire* representations covered by a transparent surface. *Right panel,* enlarged representation of the GIPR (PDB code 2QKH) and GLP-1R (PDB code 3C59) illustrating amino acid residues within 5 Å (illustrated by *ball and stick* representation) of either GIP or exendin-4, respectively. The extensions of the highlighted amino acids outside of the 5 Å distance are illustrated by *gray wire* representation.

Using the crystal structure of the isolated N-terminal GLP-1R ECD in complex with the C-terminal exendin fragment, exendin(9–39) (PDB 3C59 (5)), we have identified receptor residues within 5 Å of the peptide (Fig. 4*A*) and compared these to the receptor residues that formed cross-links with FL-Ex4 (Fig. 4*B*). Although there was significant overlap in the peptide-binding surface of the crystal structure and sites of cross-linking, including V30azF, S31azF, L32azF, V36azF, E68azF, Y69azF, L123azF, and E127azF, there was also marked divergence in the patterns of interaction. Notably, Thr-35, Trp-39, Tyr-88, Leu-89, Pro-90, Trp-91, and Arg-121 that contributed to the binding surface for exendin(9–39) each failed to cross-link to FL-Ex4 when mutated to azF. Furthermore, there was a range of azF-modified residues that formed cross-links that were not proximal to exendin(9–39) in the isolated N-terminal ECD complex: W33azF, R40azF, F80azF, Y101azF, F103azF, S117azF, and L118azF. Strong cross-linking was also observed for R134azF and to a lesser extent S135azF that are part of the TM1-ECD stalk but are absent from the isolated N-terminal ECD structure. Of note, there is a high degree of overlap in the location of ECD residues proximal to the peptide for the solved ECD structures of the GLP-1R and GIPR (Fig. 5) (5, 9), implying that lack of constraints imposed by interactions of the N-terminal ECD and receptor core may have similar impact for members of at least the GCGR subfamily.

### Generation of a GLP-1R N-terminal exendin-4 molecular model to accommodate photocross-linking constraints

**Figure 6.**
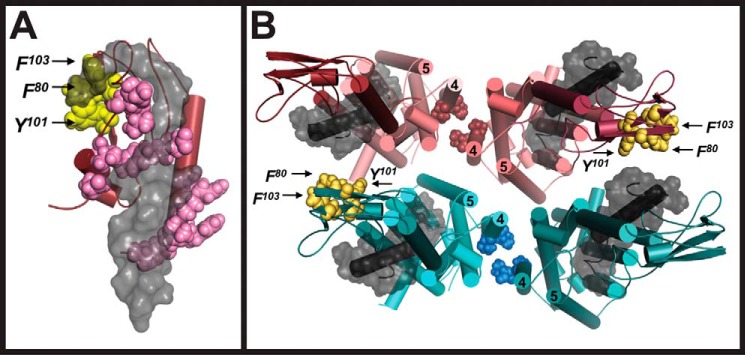
**Molecular modeling of the interaction interface between the N-terminal ECD of the human GLP-1R and exendin-4 using photocross-linking constraints.** *A,* predicted binding path of the exendin-4 peptide (*gray surface representation*) with the N-terminal ECD of the human GLP-1R (*ruby ribbon representation*). Residues positive for photocross-linking are highlighted in *pink* and *yellow* VDW representation. The C terminus of exendin-4 peptide may snake over the top of the ECD to interact with Phe-80, Tyr-101, and Phe-103 of the receptor (*yellow* VDW representation). *B,* top view of dimer of dimers model. Human GLP-1Rs form homodimers across the TM4 interface (40). Residues of TM4 involved in dimerization are highlighted in *coral* (*top*) and *cyan* (*bottom*) VDW representation. N-terminal ECDs of each receptor monomer are illustrated in *ribbon* representation in a darker shade than their corresponding TM domain color. Exendin-4 is illustrated in *gray* surface representation and potentially interacts with Phe-80, Tyr-101, and Phe-103 (*yellow* VDW representation) of the adjacent receptor dimer. The numbers *4* and *5* identify TM4 and TM5, respectively.

Using an experimentally validated GLP-1R model as a template (38, 39), a refined GLP-1R N-terminal exendin-4 model was generated to accommodate the photocross-linking data (Fig. 6*A*). This model revealed a potential for the C terminus of exendin-4 to “snake” over the top of the ECD to interact with Phe-80, Tyr-101, and Phe-103. Because an N-terminally modified FL-Ex4 was used for cross-linking, confidence that these observations were not due to altered binding of the peptide to the receptor core by the FL was achieved by comparison with an internally labeled exendin-4 peptide. Although FL(K27)-Ex4 has poor cross-linking efficiency, a specific signal could be engendered with high levels of the peptide. Under these conditions, a similar level of cross-linking to F80azF, Y101azF, and F103azF, relative to the reference Y69azF mutant, was observed for both FL(K27)-Ex4 and FL-Ex4, demonstrating that this cross-linking was not an artifact of N-terminal FL labeling of the peptide (supplemental Fig. S5). Notably, the GLP-1R also forms functionally important homodimers along the interface of TM4 (40), and as such, we also modeled receptor dimers and higher order oligomers (Fig. 6*B*). A dimer of dimers model based on a principle TM4 interface would also place the C terminus of exendin-4 in close proximity to Phe-80, Tyr-101, and Phe-103 of the adjacent dimeric receptor, making cross-dimer cross-linking a plausible explanation for the cross-linking data.

## Discussion

GPCRs all share similar structural architecture that includes seven α-helical TM domains connected by intra- and extracellular loops, an extracellular N-terminal domain, and an intracellular C-terminal domain (41). Despite these similarities, GPCRs possess unique structural and functional elements, and as such can be subdivided into different classes. In the absence of the full-length, unmodified, active receptor crystal structures for class B GPCRs, there remains significant ambiguity with respect to the mechanistic link between molecular interactions occurring at the receptor-peptide level and generation of distinct cellular responses. We begin to address this ambiguity through application of azF-mediated targeted photocross-linking to the therapeutically important class B GLP-1R, identifying interactions occurring between the receptor and an exogenous peptide in mammalian cells. This allowed for generation of molecular detail of the interactions occurring in a functionally active receptor·ligand complex in an innate setting. Although mostly analogous with published structures, several distinct differences were identified that may be important in the progression of structural and functional studies not only of the GLP-1R but also more broadly into the larger subgroup of class B receptors.

All 32 amber mutants generated in this study were trafficked to the cell surface in the presence of azF supplementation, although E125azF was not significantly different from the −azF control, and most induced a robust cAMP response in the presence of exendin-4. These data indicate that azF incorporation into the GLP-1R is generally well tolerated and that amber codon suppression technology is suitable for investigation of this highly dynamic and unstable receptor protein. The observation of expression and function of amber mutants in the absence of azF was perhaps not surprising, given that misincorporation of endogenous Tyr in the mammalian system has been documented to occur (24).

Interestingly, the peptide-binding groove for the GLP-1R, and indeed most class B GPCRs, is principally hydrophobic, and loss of direct interactions in this region would not be predicted to have major effects on peptide affinity. This is mirrored in the relatively limited impact of azF substitution on exendin-4 potency and *E*_max_ in cAMP signaling (Table 2 and supplemental Fig. S1). In all cases, reductions in *E*_max_ occurred in the absence of potency changes, indicating that there was no receptor reserve with the low levels of GLP-1R expression achieved under amber suppression. Reduced *E*_max_, in the absence of potency changes, likely reflects alterations in the presentation of the peptide N terminus to the receptor core that is critical for receptor activation. Mutations that affected exendin-4 *E*_max_ broadly fell into two categories as follows: (i) residues that reside at the extremes of the ECD (V30azF, S31azF, and L32azF at the far N terminus and R134azF and E138azF that are located near the boundary of the N-terminal ECD and TM1) consistent with peptide interactions contributing to receptor activation, and (ii) residues within the hydrophobic core of the ECD, including three Tyr residues (Y42azF, Y69azF, Y88azF, and L123azF). These Tyr residues are important for packing and tertiary structure of the ECD, with Ala mutation resulting in complete loss of function (6). AzF and Tyr are identical except for the functional group in the *para*-position of the phenyl ring, azido (−N_3_), and hydroxyl (−OH), respectively (Fig. 1*B*), and may indicate a role for H-bonding to the hydroxyl in maintenance of the optimal tertiary structure. Only four azF mutations (T35azF, A70azF, L89azF, and P90azF) resulted in loss of potency that, under the conditions of assay (no reserve), reflects decreased functional affinity for exendin-4. This could be due to effects on ligand/receptor interaction or to conformational changes to the N-terminal ECD that alter binding affinity. In accord, none of these mutants displayed cross-linking to FL-Ex4. In the GLP-1R ECD crystal structure, Ala-70 is positioned in the core of the peptide-binding domain, and it is likely that a small amino acid is required at this position to facilitate hydrophobic packing. Leu-89 is also important for ECD structure, with L89A mutation abolishing GLP-1 peptide binding (6); however, there is limited interaction between this amino acid and exendin(9–39) in the N-terminal ECD crystal structure complex, and as such the lack of cross-linking could also be due to decreased affinity for FL-Ex4, rather than lack of proximity to the peptide. This may also be true for T35azF and P90azF. T35A significantly increases exendin-4 binding, and P90A significantly decreases exendin-4-mediated signaling (6), consistent with a role for these amino acids in either peptide binding or N-terminal ECD conformation.

For all other mutants, exendin-4 potency and *E*_max_ were unaltered, suggesting that the mutations did not alter receptor conformation and that cross-linking (or absence thereof) provided specific information about the proximity of the peptide and the N-terminal ECD in an intact functional receptor. The exception was E125azF, where no significant difference in cell-surface expression was observed in the presence and absence of azF supplementation (Fig. 2*A*), indicating poor incorporation or tolerance of azF at this position. Although not explicitly reported to interact with exendin-4, Glu-125 has been noted as a putative interaction site for the GLP-1 peptide through Bpa^35^-GLP-1 photolabeling (31). In these studies, cross-linking can occur across larger distances (>10 Å) (31, 42), although the distance between Gly-35 of GLP-1 and Glu-125 in the GLP-1R-GLP-1 isolated ECD crystal structure of 23 Å is outside the proximity required for photolabeling. Mutation of Glu-125 to Ala did not significantly impact GLP-1 binding affinity or function (31, 43, 44), and thus, Glu-125 does not form an interaction with peptide that is essential for receptor function.

The isolated N-terminal ECD GLP-1R structure lacks biological context, in particular, the physical contiguity of the peptide backbone from the TM domain core (extending from TM1) and potential interactions of the far N terminus of the ECD, as have been predicted in simulations of GLP-1R-GLP-1 (38) and in models of GLP-1R activation based on activity of tethered peptides (45). Such ECD and TM core interactions are also predicted from activity of inverse-agonist Abs that bind to the ECD of the GCGR (46). Although there were major overlaps of sites of cross-linking in our study and residue proximity in the isolated ECD structure (Val-30, Ser-31, Leu-32, Val-36, Glu-68, Tyr-69, Leu-123, and Glu-127), there were clear differences, including Thr-35, Trp-39, Tyr-88, Leu-89, Pro-90, Trp-91, and Arg-121, that contributed to the binding surface for exendin(9–39) but did not cross-link, and Trp-33, Arg-40, Phe-80, Tyr-101, Phe-103, Ser-117, and Leu-118 that exhibited cross-linking but were oriented away from the peptide in the crystal structure (5).

Our data indicate that in the context of the intact receptor, the far N-terminal helix of the ECD is extended toward the receptor core with a rotation of the helix toward the peptide, and that this is accompanied by closure of the binding groove bounded by Tyr-69, Leu-123, and Glu-127 that would be consistent with the requirement for physical contiguity of the peptide backbone with the top of TM1. This could also move residues Thr-35, Trp-39, Tyr-88, Leu-89, Pro-90, and Trp-91 away from the peptide interface observed in the crystal structure, while bringing Trp-33 and Arg-40 into closer proximity. Mutation of Arg-40 to Ala resulted in a small decrease in binding affinity of GLP-1 (47), which is consistent with a potential direct interaction with peptides. Arg-134 and Ser-135 are not present in the crystal structure but are located in the TM1 stalk/ECD boundary and further support closure of the groove in the full-length receptor. Although Thr-35 may still potentially form interactions with the peptide (as discussed above), mutational data for Trp-39 are consistent with our proposed model. In the ECD crystal structure, Trp-39 is oriented away from the core domain and forms weak hydrophobic interactions with exendin(9–39) (5) that would be expected to have only limited impact on peptide affinity. In contrast, mutation of Trp-39 to Ala or Phe resulted in loss of detectable GLP-1 binding, despite maintaining expression comparable with the wild-type receptor (47, 48). Similarly, mutation of the equivalent GCGR residue Trp-36 to Ala resulted in no detectable binding of GCG, despite significantly increased expression compared with the wild-type receptor (12). These data imply that, like other key hydrophobic residues, Trp-39 is essential to ensure correct folding/conformation of the ECD, and rotation of the N-terminal ECD helix would position Trp-39 toward the core of the ECD.

The position of cross-linking residues at amino acid Ser-117 and Leu-118 is consistent with potential extension of the C terminus of exendin-4, with the N-terminal ECD GLP-1R-exendin(9–39) structure lacking density for amino acids 34–39 of the peptide (5). These results imply that this segment is relatively mobile, and the interaction of exendin-4 is readily accommodated in molecular models of the full-length receptor (Fig. 6). Interestingly, Ser-117 is part of the Asn-115/Ser-116/Ser-117 consensus glycosylation site within the GLP-1R (Fig. 3*C*) (49), with mutation predicted to disrupt glycosylation. S117azF had wild-type behavior in function, indicating that glycosylation at this site is not required for receptor expression and function, and this is consistent with the N115L mutation that had wild-type expression and function (50). Asn-115 is positioned away from the ECD core, and glycosylation at this site would be unlikely to interfere with peptide/receptor interactions.

The most intriguing finding of this study was the weak cross-linking to positions Phe-80, Tyr-101, and Phe-103 that are contiguous in 3D space but are located on the “back” face of the N-terminal ECD, away from the primary peptide-binding groove. These residues have not previously been documented to form interactions with any peptide ligand of the GLP-1R and are located in a region distal to the peptide-binding domain as determined by isolated N-terminal ECD structures (5, 6). As mentioned above, the N-terminal crystal structure of the GLP-1R·exendin complex excludes six C-terminal residues of the peptide (GAPPPS) (5). This Pro-rich fragment is fundamental to the formation of the “Trp-cage,” a compact hydrophobic “cage” arrangement encasing Trp-25 of the exendin-4 peptide. However, the conformation and dynamics of the Trp-cage is largely dependent on the environmental conditions (51–55), and in the context of a cellular setting, it may therefore potentially exist in a landscape of both a compact rigid state and a more disordered flexible state. The latter may then allow for the C terminus of the peptide to snake back over the top of the ECD to form interactions with Phe-80, Tyr-101, and Phe-103 (Fig. 6*A*). However, with the ability of the GLP-1R to form homodimers along the interface of TM4 (40), a dimer of dimers model also accounts for cross-linking between exendin-4 and Phe-80, Tyr-101, and Phe-103 of the adjacent dimeric receptor (Fig. 6*B*). Furthermore, Glu-125 is located on the same face of the ECD as Phe-80, Tyr-101, and Phe-103, and a dimer of dimers model would place this residue in a position that would be compatible with the Bpa-mediated cross-linking with position 35 of the GLP-1 peptide (31), as discussed above. Notably, GLP-1 is shorter than exendin-4 and would not be able bridge the distance by snaking of the C terminus toward the top of the ECD.

In this study, we have used the powerful technique of receptor-based targeted photocross-linking to investigate the molecular interactions occurring between the exendin-4 peptide and the N-terminal ECD of the full-length, fully functional human GLP-1R in mammalian cells, thereby simulating a native receptor environment. Our data provide novel insights into the dynamics of peptide interaction with the receptor that contribute to linking receptor structure with molecular function and the pursuit of structure-based drug design.

## Experimental procedures

### Materials

DMEM, PBS, Hanks' buffered salt solution (HBSS), Lipofectamine^TM^ reagent, PLUS^TM^ reagent, NuPAGE® 4–12% BisTris protein gels and buffers, DynaBeads® protein G, chemiluminescent substrate, and monoclonal mouse anti-V5 Ab (R960-25) were purchased from Life Technologies, Inc. Polyclonal rabbit anti-FL Ab (ab19491) was purchased from Abcam (Cambridge, MA). The QuikChange® Lightning site-directed mutagenesis kit was purchased from Stratagene (La Jolla, CA). AzF was purchased from Chem Impex International (Wood Dale, IL). Exendin-4 was purchased from American Peptide (Sunnyvale, CA). FL-labeled exendin-4 analogues were purchased from Anaspec (Fremont, CA) and NeoScientific (Cambridge, MA). AlphaScreen^TM^ reagents and 384-well ProxiPlates were purchased from PerkinElmer Life Sciences. SigmaFast^TM^ *o*-phenylenediamine dihydrochloride tablets, *n*-dodecyl β-d-maltoside (DM), and monoclonal mouse anti-c-Myc Ab (M4439, clone 9E10) were purchased from Sigma. PVDF membranes were purchased from EMD Millipore (Billerica, MA). HyBlot CL autoradiography film was purchased from Denville Scientific (Holliston, MA). FBS was purchased from Gemini Bioproducts (West Sacramento, CA). All other reagents were obtained from Thermo Fisher Scientific (Waltham, MA) and were of analytical grade.

### Constructs and receptor mutagenesis

Suppressor tRNA and azF amino-acyl tRNA synthetase plasmids were constructed as described previously (24). Amber codons (TAG) were introduced in the desired location of an N-terminal double c-Myc-labeled wild-type human GLP-1R in the pEF5/FRT/V5-DEST destination vector (Invitrogen). Endogenous amber codons in this construct were mutated to opal codons (TGA), and this receptor had equivalent pharmacology to the unmodified human GLP-1R (data not shown). Mutagenesis was carried out using oligonucleotides for site-directed mutagenesis purchased from Thermo Fisher Scientific and the QuikChange® Lightning site-directed mutagenesis kit (Stratagene) and was confirmed by automated sequencing.

### Transfections and cell culture

HEK293T cells were transiently transfected in serum-free DMEM using Lipofectamine^TM^/PLUS^TM^. Wild-type human GLP-1R, suppressor tRNA and azF amino-acyl tRNA synthetase were typically transfected at a ratio of 1:10:5 (μg), whereas the amber mutant human GLP-1R, suppressor tRNA, and azF amino-acyl tRNA synthetase were typically transfected at a ratio of 2:2:1 (μg). In all cases, the combined total of DNA per well was 2.5 μg (6-well), 625 ng (24-well), or 100 ng (96-well). Four hours post-transfection, transfected cells were supplemented with serum-enriched DMEM for a final concentration of 10% (v/v) FBS with or without 0.5 mm azF.

### Photocross-linking

HEK293T cells were seeded at 8 × 10^5^ cells/well in 6-well culture plates, transiently transfected using Lipofectamine^TM^/PLUS^TM^ as detailed above, and incubated for 24 h at 37 °C in 5% CO_2_. 24 h post-transfection, media were replaced with 10 or 100 nm FL-conjugated exendin-4 analogues (Anaspec, Neoscientific) with or without increasing concentrations of unlabeled exendin-4, each prepared in HBSS, 0.1% (w/v) BSA, 20 mm HEPES under dim light conditions, and incubated overnight at 4 °C.

Following incubation, cells were irradiated with a Maxima ML-3500S UV-A light (Spectronics Corp.) for 2 min at 4 °C on ice. Cells were then centrifuged at 850 × *g* for 5 min, and the pellet was resuspended in solubilization buffer (Tris-HCl, pH 7.5, 1% DM). Solubilization was carried out for 1 h at 4 °C on a nutating mixer (Thomas Scientific) followed by centrifugation at 10,000 × *g* for 15 min at 4 °C to isolate proteins (supernatant fraction) from cellular debris (pellet).

### Immunoprecipitation

Wild-type human GLP-1R or amber mutant GLP-1Rs, labeled with a C-terminal V5 epitope, were immunopurified from detergent-solubilized cell lysates using Dynabeads® protein G (Life Technologies, Inc.) complexed with mouse anti-V5 mAb (Life Technologies, Inc.) according to the manufacturer's instructions. Briefly, Dynabeads® protein G were complexed with anti-V5 for 10 min at room temperature, followed by incubation with cell lysates for 10 min at room temperature. V5-labeled proteins were eluted using 50 mm glycine, pH 2.8, for 10 min at room temperature.

### Immunoblotting

Immunopurified proteins were mixed with 4× NuPAGE® LDS Sample Buffer (Life Technologies, Inc.) and DTT (100 mm) prior to separation on NuPAGE® 4–12% BisTris gels (Life Technologies, Inc.) using NuPAGE® MES SDS Buffer (Life Technologies, Inc.) with NuPAGE® antioxidant (Life Technologies, Inc.) at 160 V for ∼70 min on ice. Gel-separated proteins were transferred to PVDF membranes (EMD Millipore) at 18 V for 45 min at room temperature using a semidry transfer apparatus (Bio-Rad). Membranes were incubated in blocking buffer (5% (w/v) nonfat dry milk in TBS-T (10 mm Tris-HCl, 150 mm NaCl, 0.05% (v/v) Tween 20, pH 7.4)) for 2 h at room temperature with gentle agitation, followed by primary Ab (diluted in blocking buffer 1:3000 for anti-FL and 1:5000 for anti-V5) overnight at 4 °C on a nutating mixer (Thomas Scientific). The following day, membranes were washed with TBS-T and incubated with HRP-linked secondary Ab (diluted in blocking buffer 1:10,000) for 1 h at room temperature with gentle agitation. Membranes were again washed with TBS-T, treated with chemiluminescent substrate (Thermo Fisher Scientific) for 3 min at room temperature, and exposed to HyBlot CL autoradiography film (Denville Scientific). Densitometry analysis of immunoblots was performed using ImageJ 1.48 software (National Institutes of Health). Relative cross-linking of FL-labeled exendin-4 peptides to receptor mutants was determined as a measure of optical density, normalized to the respective controls on the same blot.

### cAMP accumulation assay

HEK293T cells were seeded at 2 × 10^4^ cells/well in 96-well poly-d-lysine (PDL)-coated culture plates, transiently transfected using Lipofectamine^TM^/PLUS^TM^ as detailed above, and incubated for 48 h at 37 °C in 5% CO_2_. Ligand-mediated cAMP generation was then carried out as described previously (35). All values were converted to concentration of cAMP using a cAMP standard curve performed in parallel, and data were subsequently normalized to the response of 10 μm forskolin at each receptor mutant.

### Cell-surface receptor expression

HEK293T cells were seeded at 2 × 10^5^ cells/well in 24-well PDL-coated culture plates, transiently transfected using Lipofectamine^TM^/PLUS^TM^ as detailed above, and incubated for 48 h at 37 °C in 5% CO_2_. Cells were washed three times in PBS and fixed with 4% paraformaldehyde for 15 min at 4 °C. Cell-surface receptor expression was then measured as described previously (56). All data were normalized to the basal fluorescence detected in mock-transfected HEK293T cells (pcDNA, tRNA suppressor, and azF amino-acyl tRNA synthetase only).

### Data analysis

All data were analyzed using Prism 6.0 (GraphPad Software Inc., San Diego). For all analyses, the data were unweighted, and each *y* value (mean of replicates for each individual experiment) was considered an individual point. Concentration-response signaling data were analyzed using Equation 1, as described previously (57),

(Eq. 1) $$\text{Y}=\text{bottom}+\frac{\left(\text{top}-\text{bottom}\right)}{1+10^{\left(\log{\text{EC}}_{50}-\log\left[A\right]\right)}}$$

where bottom represents the *y* value in the absence of ligand(s); top represents maximal stimulation in the presence of ligand(s); [*A*] is the molar concentration of ligand, and *EC*_50_ is the molar concentration of ligand required to generate a response halfway between top and bottom. AzF-dependent cell-surface expression of amber mutants was determined by correcting for expression measured in the absence of azF. Accordingly, azF-dependent expression included propagation of errors from both −azF and +azF conditions.

### Statistics

Changes in total cell-surface expression for each mutant in the absence and presence of azF was determined using an unpaired *t* test, and statistical significance accepted as *p* < 0.05. Changes in peptide potency, efficacy, and azF-dependent cell-surface expression of azF-incorporated human GLP-1R mutants in comparison to wild-type human GLP-1R control were statistically analyzed with one-way analysis of variance and Dunnett's post-test, and statistical significance accepted as *p* < 0.05.

### Molecular modeling

#### 

##### Modeling the human GLP-1R in complex with exendin

The intact active-state model of the human GLP-1R in complex with exendin-4 and the C-terminal helix of Gα_s_ was modeled using a refined and experimentally validated model of the GLP-1R in complex with GLP-1 as a template (38, 39). One hundred models were generated with Modeler (58), and the best ranked models were selected with the discrete optimized protein energy scoring function (59) and visually inspected. The selected model was embedded in a lipidic bilayer and simulated for 0.5 μs as described under “Molecular Dynamics Simulations.”

##### Molecular dynamics simulations

Simulations were prepared as described previously (38, 39). Briefly, the full active-state model of the human GLP-1R in complex with exendin-4 was immersed in a lipidic bilayer of palmitoyl oleoyl phosphatidylcholine with explicit water and ions at a concentration of 150 mm and simulated using ACEMD (60), the AMBER 14SB (61), and lipid 14 force fields (62). The system was composed of 92,776 atoms, and the starting system dimensions were 85 × 86 × 138 Å. The simulation procedure was composed of steps of energy minimization, heating from 0 to 300 K, and a progressive decrease of conformational constraints followed by the unconstrained production run over 0.5 μs.

##### Modeling of a TM4-TM4 dimer and a dimer of dimers of the human GLP-1R

Two monomers of the active-state human GLP-1R receptor bound to exendin-4 were modeled as a homodimer with a TM4/TM4 interface, with the assistance of the graphical user interface (GUI) of Maestro (Schrödinger LLC). The modeled dimer satisfies experimental constraints since the residues Gly-275, Leu-279, and Val-282, which were reported to disrupt the GLP-1R dimer formation when mutated (40), are oriented and clustered facing one another at the interface of the dimer (Fig. 6*B*). This dimeric model of the GLP-1R was used as a search unit for a novel dimer of dimers interface that would satisfy the cross-linking data for residues on the back side of the N-terminal ECD. Using the GUI, a new dimer of dimers arrangement that satisfies the constraints of the cross-linking results was identified.

## Author contributions

C. K., P. M. S., and T. P. S. designed the study and wrote or contributed to writing of the manuscript. C. K. conducted cAMP, ELISA, and all cross-linking experiments and performed data analysis and interpretation. C. H. conducted cAMP and ELISA experiments. C. A. R. and J. C. M. generated molecular models. All authors reviewed the results and approved the final version of the manuscript.

## Supplementary Material

Supplemental Data

## Footnotes

4 The abbreviations used are:

GPCR: G protein-coupled receptor
Ab: antibody
azF: *p*-azido-l-phenylalanine
Bpa: *p*-benzoyl-l-phenylalanine
CRF: corticotropin-releasing factor
DM: *n*-dodecyl β-d-maltoside
ECD: extracellular domain
FL: fluorescein
FL-Ex4: fluorescein-exendin-4
FL(K20)-Ex4: fluorescein(Lys-20)-exendin-4
FL(K27)-Ex4: fluorescein(Lys-27)-exendin-4
FL(W25)-Ex4: fluorescein(Trp-25)-exendin-4
GCG: glucagon
GCGR: glucagon receptor
GIP: gastric inhibitory polypeptide
GIPR: gastric inhibitory polypeptide receptor
GLP-1R: glucagon-like peptide-1 receptor
GUI: graphical user interface
HBSS: Hanks' buffered salt solution
PDL: poly-d-lysine
TM: transmembrane
VDW: van der Waals
BisTris: 2-[bis(2-hydroxyethyl)amino]-2-(hydroxymethyl)propane-1,3-diol
PDB: Protein Data Bank.
