# Supplementary material for: Genetically encoded photocross-linkers determine the biological binding site of exendin-4 peptide in the N-terminal domain of the intact human glucagon-like peptide-1 receptor (GLP-1R)
Source: J Biol Chem. 2017 Mar 10;292(17):7131–44. doi: 10.1074/jbc.M117.779496 (PMC5409479; doi:10.1074/jbc.M117.779496)
Supplement: Supplemental Data [file supp_292_17_7131__index.html]

Genetically-Encoded Photocrosslinkers Determine the Biological Binding Site of Exendin-4 in the N-Terminal Domain of the Intact Human Glucagon-Like Peptide-1 Receptor (GLP-1R) — Genetically encoded photocross-linkers determine the biological binding site of exendin-4 peptide in the N-terminal domain of the intact human glucagon-like peptide-1 receptor (GLP-1R) — Photocross-linking maps exendin-4 binding on intact GLP-1R — Supplemental Data 

# Genetically encoded photocross-linkers determine the biological binding site of exendin-4 peptide in the N-terminal domain of the intact human glucagon-like peptide-1 receptor (GLP-1R)

## Supplemental Data

- Supplementary Figures (.pdf, 6.4 MB) - Supplementary Figures, minor change to legend of SI Figure 3 as per reviewers comments
