# Supplementary material for: Genetically encoded photocross-linkers determine the biological binding site of exendin-4 peptide in the N-terminal domain of the intact human glucagon-like peptide-1 receptor (GLP-1R)
Source: J Biol Chem. 2017 Mar 10;292(17):7131–44. doi: 10.1074/jbc.M117.779496 (PMC5409479; doi:10.1074/jbc.M117.779496)
Supplement: Supplemental Data [file 10.1074_M117.779496_jbc.M117.779496-1.pdf]

SI Figure S1.

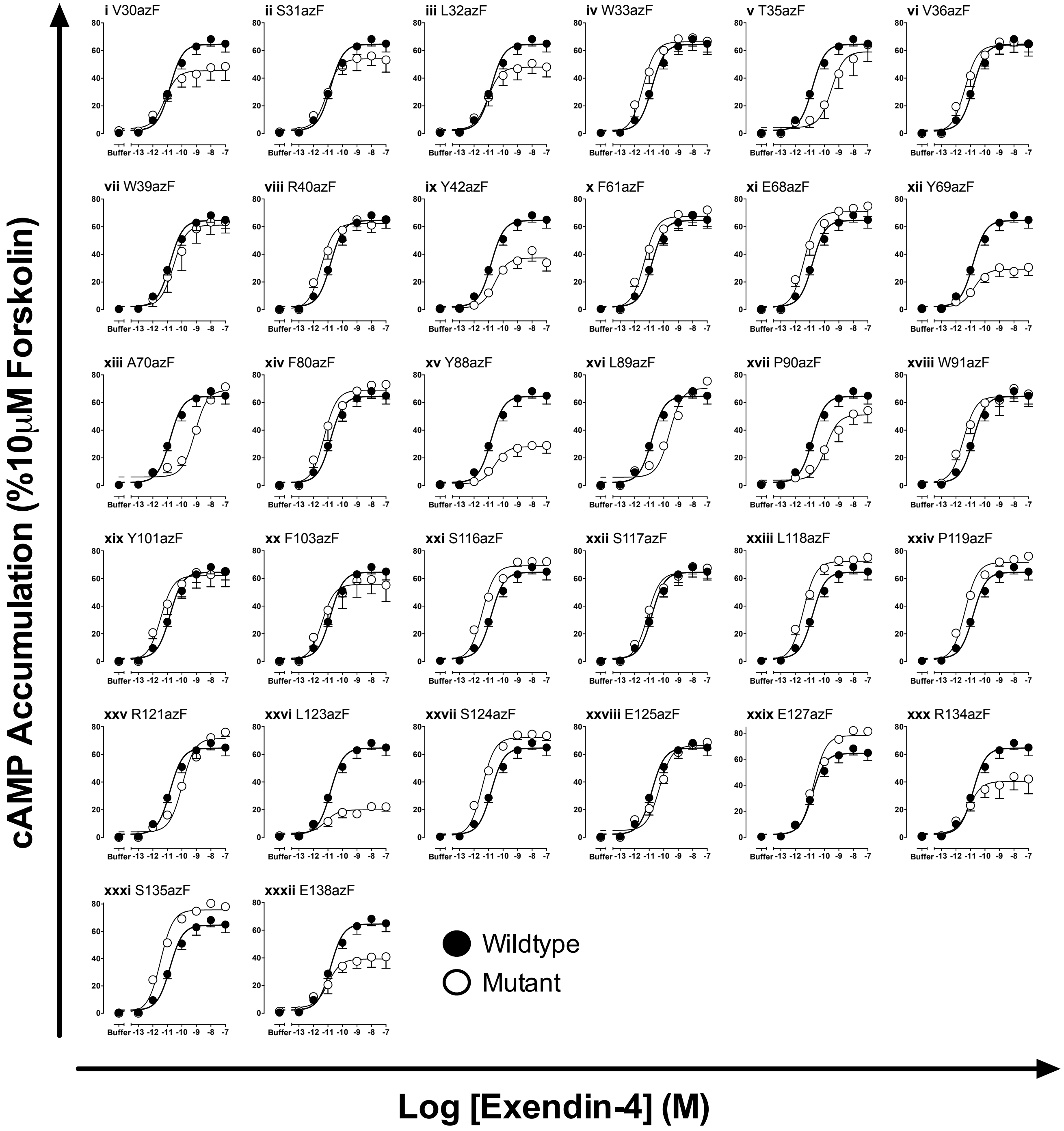

Supplementary Figure S1. ***Exendin-4-Mediated cAMP Accumulation at azF-Incorporated Human GLP-1Rs.*** Characterization of exendin-4-mediated cAMP accumulation in HEK293T cells transiently expressing the wildtype human GLP-1R (●) or each of the azF-incorporated GLP-1Rs (○), in the presence of 0.5 mM azF. Wildtype human GLP-1R was transfected at one-tenth that of mutants. Data are normalized to the response elicited by 10 μM forskolin and analyzed using a three-parameter logistic equation as defined in Equation 1. All values are mean ± S.E.M. of three to five independent experiments, conducted in duplicate.

SI Figure S2.

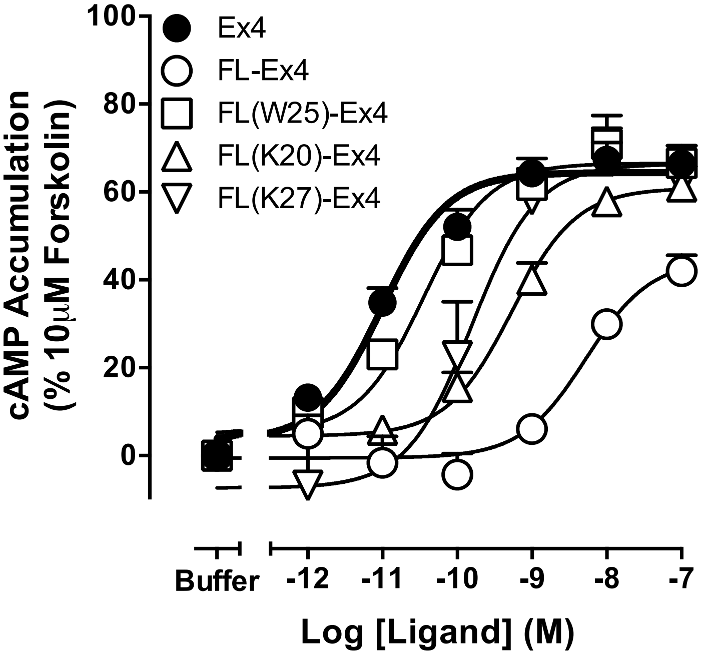

Supplementary Figure S2. ***cAMP Accumulation Profiles of Fluorescein (FL)-Labeled Exendin-4 (Ex4) Analogues at the Wildtype Human GLP-1R.*** Characterization of cAMP accumulation in HEK293T cells transiently expressing the wildtype human GLP-1R in the presence of Ex-4 (●), FL-Ex4 (○), FL(W25)-Ex4 (□), FL(K20)-Ex4 (△) and FL(K27)-Ex4 (▽). Data are normalized to the response elicited by 10 μM forskolin and analyzed using a three-parameter logistic equation as defined in Equation 1. All values are mean ± S.E.M. of three to six independent experiments, conducted in duplicate.

SI Figure S3.

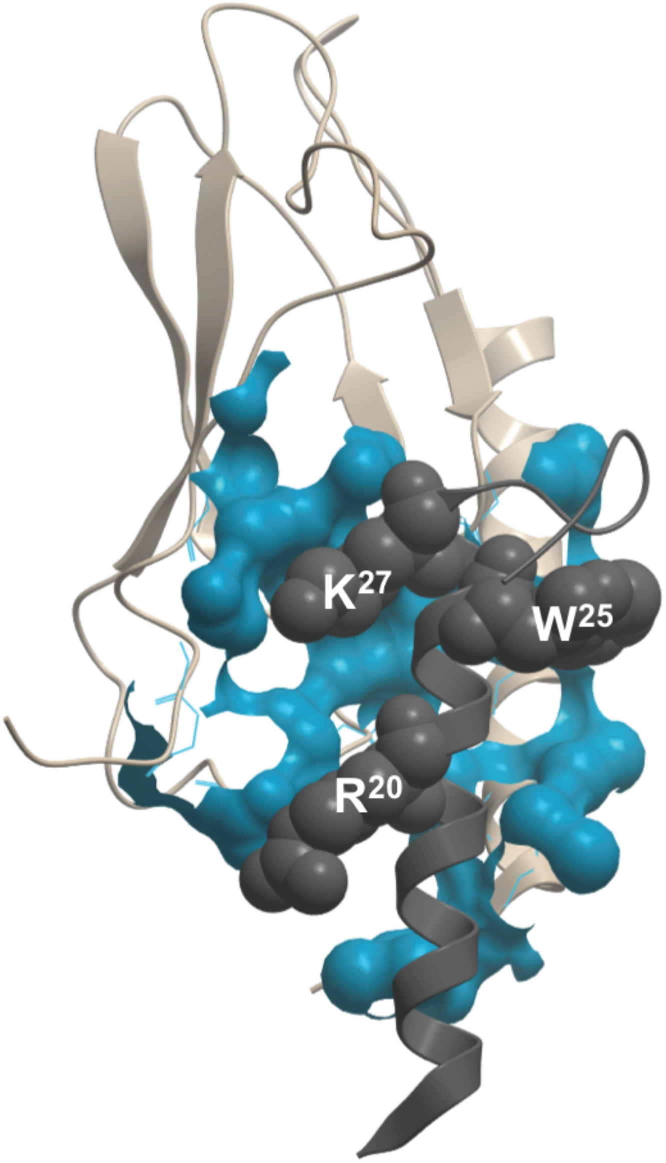

Supplementary Figure S3. ***Positions of Fluorescein (FL) Conjugation to Exendin-4 in Relation to the Documented N-Terminal Interaction Interface of the Human GLP-1R.*** Crystal structure of the isolated N-terminus of the human GLP-1R in complex with exendin-4 (PDB: 3C59), illustrating the position of mid-chain residues used for FL conjugation (CPG representation, dark grey); Arg20 was substituted with Lys for labeling. The interaction surface of the receptor (within 5 Å of exendin-4) is shown in blue surface representation, the backbone of the receptor N-terminus in off-white ribbon and the backbone of the exendin-4 peptide in dark grey ribbon.

SI Figure S4.

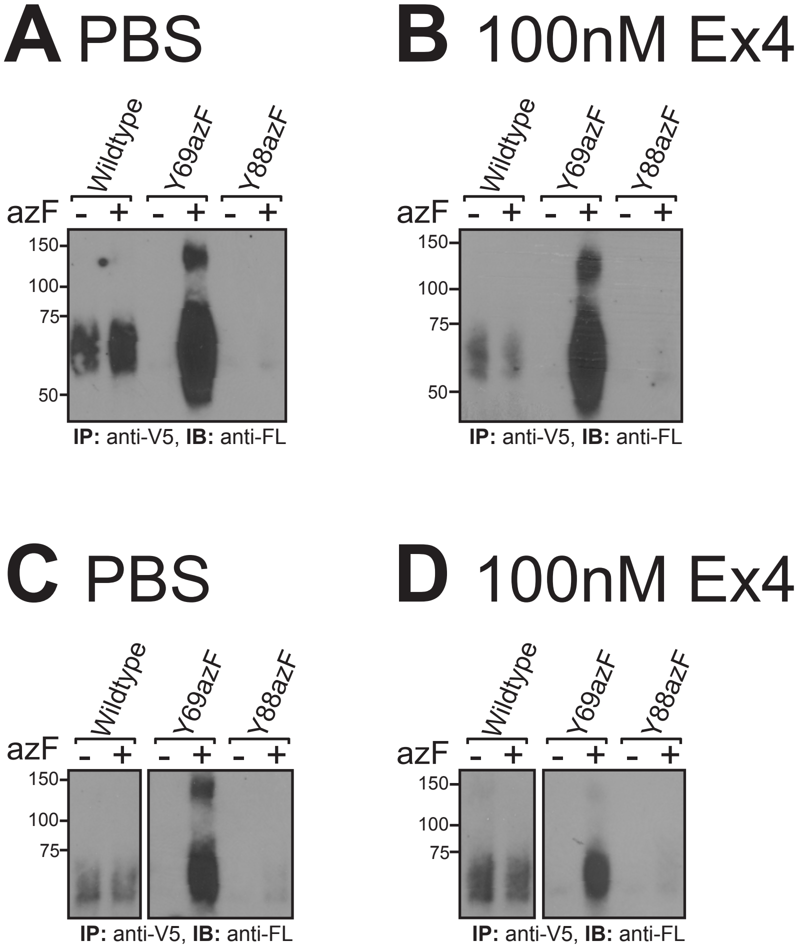

Supplementary Figure S4. ***UV-Mediated Crosslinking between azF-Incorporated Human GLP-1Rs and Fluorescein (FL)-Labeled Exendin-4 (Ex4) is Irreversible*****.** HEK293T cells transiently expressing human GLP-1R wildtype or amber mutants in the absence (-) and presence (+) of 0.5 mM azF were incubated with **(A,B)** 10 nM FL-Ex4 or **(C,D)** 100 nM FL(K27)-Ex4, followed by exposure to UV light for 2 min at 4°C. Cells were then incubated with **(A,C)** PBS or **(B,D)** 100 nM unlabeled Ex4 followed by lysis. Whole cell lysates were immunoprecipitated (IP) using an anti-V5 Ab to isolate full length GLP-1Rs, and products resolved by SDS-PAGE. Bands detected with the anti-FL Ab (immunoblot, IB) identify receptor positions at which azF covalently captures the FL-labeled exendin-4 ligand. Some background crosslinking was observed between FL-labeled exendin-4 peptides and the wildtype human GLP-1R. Data are representative of two independent experiments.

SI Figure S5.

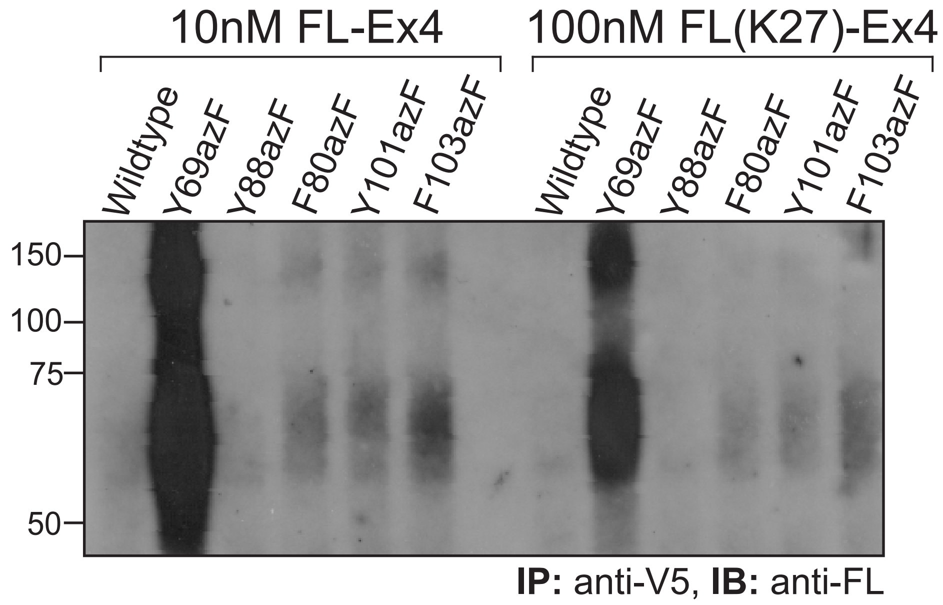

Supplementary Figure S5. ***Location of Fluorescein (FL) Conjugation to the Exendin-4 (Ex4) Peptide does not Impact the UV-Mediated Crosslinking Profile at azF-Incorporated Human GLP-1Rs.*** HEK293T cells transiently expressing human GLP-1R wildtype or amber mutants in the presence of 0.5 mM azF were incubated with 10 nM FL-Ex4 or 100 nM FL(K27)-Ex4 followed by exposure to UV light for 2 min at 4°C. Whole cell lysates were then immunoprecipitated (IP) using an anti-V5 Ab to isolate full length GLP-1Rs, and products resolved by SDS-PAGE. Bands detected with the anti-FL Ab (immunoblot, IB) identify receptor positions at which azF covalently captures the FL-labeled exendin-4 ligand. Data are representative of three independent experiments.
